# Association between waist-hip ratio and coronary artery calcification in postmenopausal women

**DOI:** 10.1097/GME.0000000000001581

**Published:** 2020-05-22

**Authors:** Youngmi Eun, Su Nam Lee, Jin Jung, Min Sik Kim, Keon-Woong Moon, Ki-Dong Yoo

**Affiliations:** 1 Department of Family Medicine, St. Vincent's Hospital, The Catholic University of Korea, Seoul, South Korea; 2 Division of Cardiology, Department of Internal Medicine, St. Vincent's Hospital, The Catholic University of Korea, Seoul, South Korea.

**Keywords:** Coronary artery calcification, Postmenopause, Waist-hip ratio

## Abstract

**Objective:**

Many studies have reported that body composition might be associated with cardiovascular disease, but the issue has not been fully investigated in postmenopausal women.

**Methods:**

This retrospective study comprised 582 postmenopausal women without a history of cardiovascular disease who visited the Health Promotion Center between May 2008 and February 2018. All women were screened for body fat composition by bioelectrical impedance analysis and for degree of coronary artery calcification (CAC) by multidetector computed tomography. In addition, multivariate analysis, integrated discrimination improvement, and category-free net reclassification improvement were performed.

**Results:**

The level of triglycerides, and the waist-hip ratio (WHR) in participants with CAC (coronary artery calcium score [CACS] > 0) were higher than in participants with a CACS of zero points. When the participants were stratified into four groups according to WHR, participants with CAC (CACS > 0) increased significantly as WHR quartile increased. A multivariate analysis showed that older age (odds ratio [OR]: 2.539; 95% confidence interval [CI]: 1.524-4.230; *P* < 0.001), triglyceride level (OR: 1.005; 95% CI: 1.002-1.008; *P* = 0.003), WHR (OR: 1.103; 95% CI: 1.018-1.195; *P* = 0.017), and history of hypertension (OR: 2.701; 95% CI: 1.715-4.253; *P* < 0.001) were significantly associated with CAC. The Brier score upon adding WHR to a clinical model was lower than that of the clinical model without WHR. Adding WHR to a clinical model better predicted CAC than a clinical model without WHR (C index: 0.761, 95% CI: 0.724-0.795, *P* < 0.001; net reclassification improvement: 0.195, *P* = 0.037; integrated discrimination improvement: 1.02%, *P* = 0.043).

**Conclusions:**

In asymptomatic postmenopausal women, WHR as measured by bioelectrical impedance analysis was significantly associated with coronary atherosclerosis, supplementing information of usual clinical markers. Hence, WHR might be appropriate as a marker for early atherosclerosis.

Obesity is strongly related to cardiovascular disease (CVD) and has increased exponentially over the past few decades worldwide.^1,2^ In Korea in particular, the prevalence of obesity increased from 26% in 1998 to 34.1% in 2017.^3^ The relationship between obesity and CVD depends not only on the total amount of body fat but also on body fat composition.^4,5^ Furthermore, women indispensably undergo adverse changes in body fat composition, lipids, and vascular remodeling during menopause.^6-10^

Early detection of subclinical atherosclerosis is important to prevent CVD.^11^ Coronary artery calcification (CAC) is a surrogate marker of total coronary atherosclerotic burden. The coronary artery calcium score (CACS) is a reliable tool for estimating risk of CVD.^12,13^

Previous studies have reported an association between body fat composition and CAC in healthy participants.^5^ Such an association has, however, not yet been reported in postmenopausal women. The aim of this study was to investigate the relationship between body fat composition, as assessed by bioelectrical impedance analysis (BIA), and CAC as assessed by multidetector computed tomography in asymptomatic postmenopausal women.

## METHODS

### Study population

In total, 687 postmenopausal women undergoing coronary computed tomography angiography and BIA, who visited the Health Promotion Center of St. Vincent's Hospital, the Catholic University of Korea, Republic of Korea, from May 2008 to February 2018 were enrolled in this retrospective, observational study. Patients with angina (*n* = 63), myocardial infarction (*n* = 23), and stroke (*n* = 19) were excluded. Thus, a total of 582 postmenopausal women were included in this study. The current study was exempted from the need to procure written informed consent because the involved medical data were reviewed retrospectively. All data records were anonymously identified and analyzed. This study was approved by the Institutional Review Board (IRB) of St. Vincent's Hospital, The Catholic University of Korea (IRB approval no: VC20RISI0037).

### Clinical and laboratory measurements

Anthropometric measurements were performed in duplicate, and the results were averaged by specially trained examiners. Height and weight were measured after an overnight fast, whereas body mass index was calculated as participant weight in kilograms divided by the square of height in meters. Blood pressure measurements were performed with a standardized sphygmomanometer in the sitting position after 10 minutes of rest. Waist circumference was measured at the midpoint between the lowest rib and the anterior iliac crest in the standing position. All participants completed a questionnaire that solicited information regarding age, smoking status, menopause, and medical history. Smoking history was defined as a current smoker or exsmoker. Menopause was defined as lack of menstruation for 1 full year. History of hypertension, diabetes mellitus (DM), or dyslipidemia was defined as a previous diagnosis before the current examination and use of antihypertensive, blood glucose, or lipid-lowering agents, respectively.

After a 12-hour fast, blood samples were collected from the antecubital vein of all participants. Total cholesterol, triglycerides, low-density lipoprotein cholesterol, high-density lipoprotein cholesterol, and creatinine levels were determined using a Hitachi 747 autoanalyzer (Hitachi, Tokyo, Japan).

### Assessment of body fat composition

Body composition measurements, of total body fat mass (kg), skeletal muscle mass (kg), waist-hip ratio (WHR), and visceral fat area (VFA, cm^2^) were estimated using a multifrequency BIA (InBody 720, Biospace Co., Seoul, Korea) by two trained examiners. The fat mass index was calculated as follows: total body fat (kg)/height^2^ (m). The sum of muscle mass of the four limbs was defined as appendicular skeletal muscle mass (ASM).

### Coronary artery calcium score

Assessment of the coronary arteries was measured using 64-slice multidetector computed tomography (Sensation 64; Siemens, Erlangen, Germany) and recorded by two trained technicians. Assessment of the coronary arteries has been described in detail in a previous study.^14^ The presence of CAC was defined by a CACS > 0 and was further subdivided into 0 < CACS < 100 and CACS ≥100. Intermediate CACS was defined as an Agatston score of 0 to 100. High CACS was defined as an Agatston score ≥100.

### Statistical analysis

Continuous variables are expressed as median and interquartile range or mean ± standard deviation and compared using analysis of variance, followed by a post-hoc test using the multiple comparison—Bonferroni method. Categorical variables are presented as numbers and percentages and were compared using the chi-square or Fisher exact test. Receiver operating characteristic (ROC) curves were analyzed to assess the cut-off point of WHR for the prediction of CAC. A logistic regression analysis was conducted to determine independent predictors associated with CAC. Variables with significant associations in a univariate analysis (*P* < 0.05) were entered into a multivariate analysis. Odds ratios (ORs) and 95% confidence intervals (CIs) were also calculated. In univariate analysis, we identified significant variables (*P* < 0.05), examined correlation and multicollinearity, and selected the final variables in the clinical model. The clinical model included age, triglycerides, fat mass index, ASM/height^2^, skeletal muscle mass, visceral fat area, smoking, DM, hypertension, and dyslipidemia. The Brier scores of this clinical model and that with addition of WHR were also calculated. The Brier score is a proper score function that measures the accuracy of probabilistic predictions and the lower the Brier score means that the better predictions are calibrated. We additionally used absolute integrated discrimination improvement and category-free net reclassification improvement to evaluate improvements in risk prediction. A *P* value less than 0.05 was considered statistically significant. All analyses were performed using Statistical Analysis Software version 9.4 (SAS Institute, Cary, NC).

## RESULTS

### Baseline characteristics of the study population

**FIG. 1:**
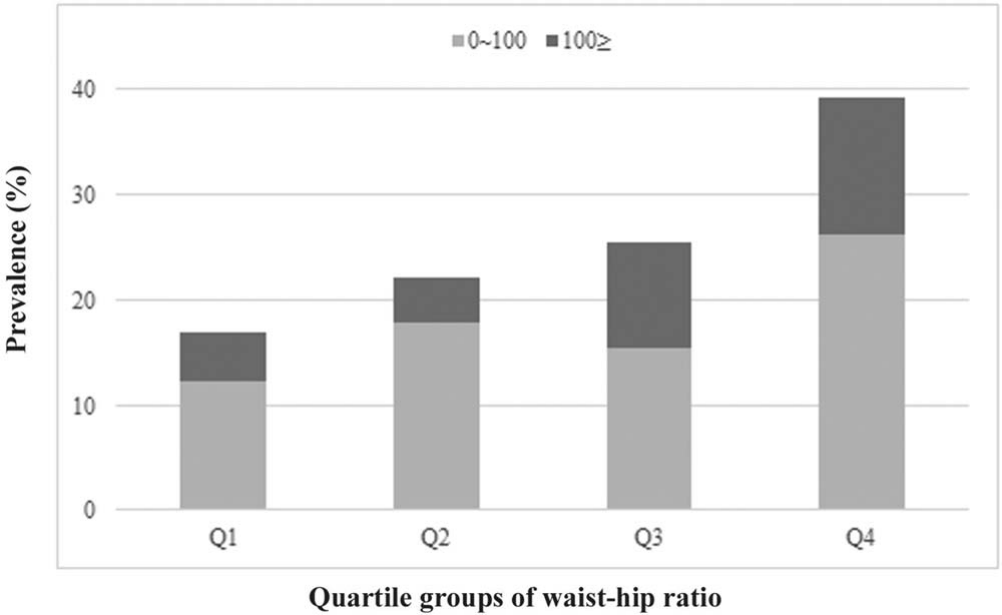
Prevalence of participants with coronary artery calcification (CACS > 0) according to quartile of waist-hip ratio.

The baseline patient characteristics are listed in Table 1. Among the 582 postmenopausal women, the proportions of intermediate CACS and high CACS were 18.4% and 8.4%, respectively. Mean age, prevalence of DM, hypertension, and dyslipidemia, level of HbA1C, and VFA increased significantly with CAC severity. In addition, level of triglycerides and WHR in participants with CAC (CACS > 0) were higher than in participants with CACS of 0. The levels of total cholesterol and low-density lipoprotein–cholesterol in intermediate CACS were higher than in the other two groups. Skeletal muscle mass in participants with CAC (CACS > 0) was, however, lower than in participants with CACS of 0. When the participants were stratified into four groups according to WHR, the proportion of participants with CAC (CACS > 0) increased significantly as WHR quartile increased (Fig. 1).

**TABLE 1:**
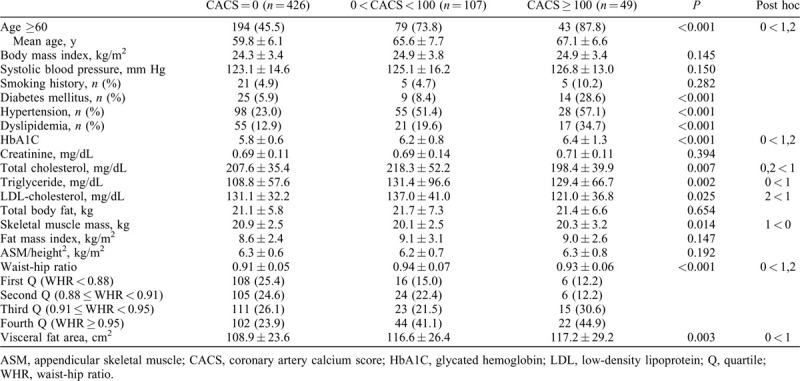
Baseline characteristics

### Predictive value of waist-hip ratio for coronary artery calcification

ROC analysis demonstrated that WHR (area under the ROC curve: 0.614: 95% CI: 0.573-0.654, *P* < 0.001) was a significant predictor of CAC. The optimal cut-off value was WHR > 0.94. At this value, sensitivity and specificity were 42.3% and 76.1%, respectively.

### Predictors of coronary artery calcification

A multivariate analysis showed that older age (OR: 2.539; 95% CI: 1.524-4.230; *P* < 0.001), triglycerides (OR: 1.005; 95% CI: 1.002-1.008; *P* = 0.003), WHR (OR: 1.103; 95% CI: 1.018-1.195; *P* = 0.017), and history of hypertension (OR: 2.701; 95% CI: 1.715-4.253; *P* < 0.001) were significantly associated with CAC (Table 2).

**TABLE 2:**
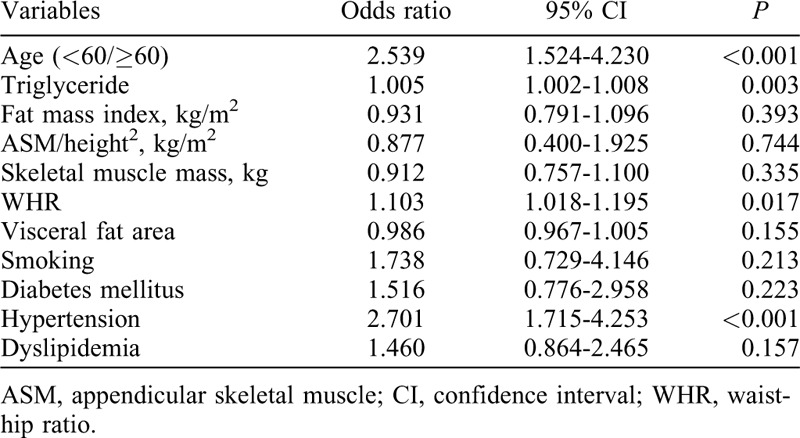
Multivariate logistic analysis

### Predictive performance of waist-hip ratio for the coronary artery calcification prediction

**FIG. 2:**
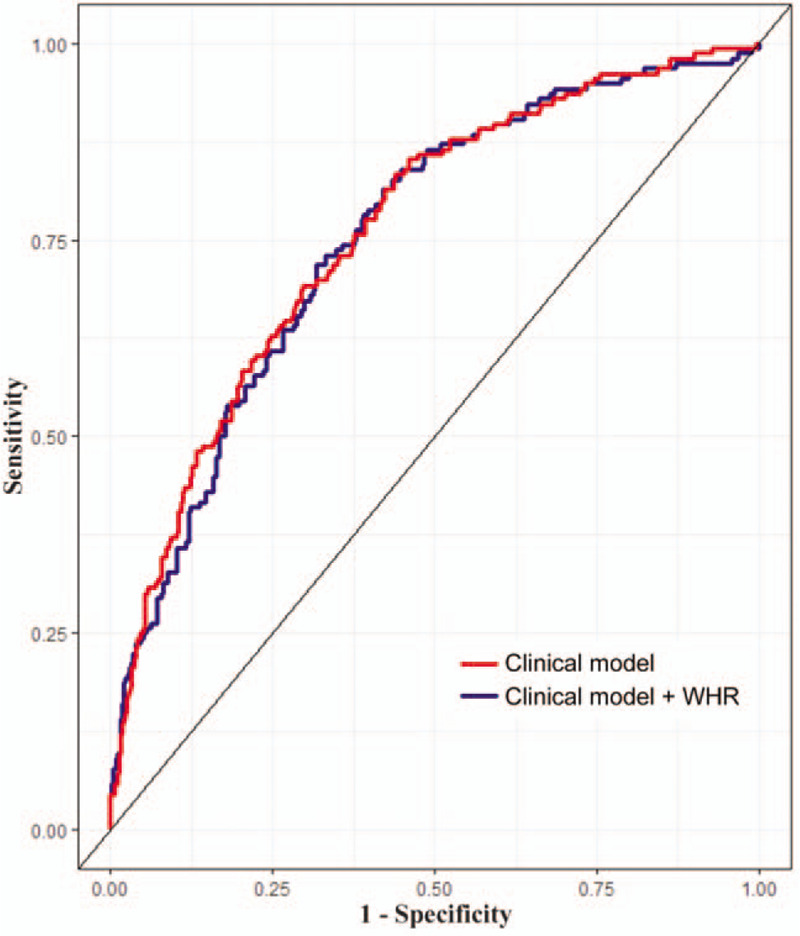
Receiving operator characteristic curves of a clinical model and of that model with addition of waist-hip ratio for coronary artery calcification. WHR, waist-hip ratio.

The clinical model included age, triglycerides, fat mass index, ASM/height^2^, skeletal muscle mass, VFA, smoking, DM, hypertension, and dyslipidemia. The Brier score when adding WHR to this clinical model was lower than that of the clinical model without WHR (Table 3). Furthermore, the prognostic performance when adding WHR to the clinical model was better than that of the clinical model without WHR (C index: 0.761, 95% CI: 0.724-0.795, *P* < 0.001; net reclassification improvement: 0.195, *P* = 0.037; integrated discrimination improvement: 1.02%, *P* = 0.043) (Table 3 and Fig. 2).

**TABLE 3:**
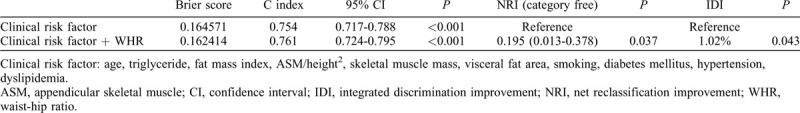
Net reclassification improvement analysis and integrated discrimination improvement

## DISCUSSION

In this study, we evaluated the relationship between body fat composition and coronary atherosclerosis in asymptomatic postmenopausal women. Higher WHR showed an increased prevalence for CAC, and WHR was significantly associated with CAC. Adding WHR to a clinical model improved prediction of CAC. To the best of our knowledge, this is the first study to investigate the relationship between body fat composition and CAC in postmenopausal women.

A relationship between body composition and CAC has been reported in the general population.^5,15^ Yu et al^5^ investigated the association between CACS and body composition in healthy adults and showed that WHR was the most significant predictor of CAC after adjusting for confounding factors. Most of the female participants in their study were, however, premenopausal, so the degree of CAC might have been very low. As such, the association between WHR and CAC is not traditionally clearly defined in postmenopausal women. Ko et al^15^ conducted a cross-sectional study of 31,108 asymptomatic adults to investigate whether muscle mass is related to CAC, concluding that low muscle mass was associated with prevalence of CAC. In our study, muscle mass in participants with CAC (CACS > 0) was also lower than that in participants with CACS of 0; however, this relationship was attenuated after adjusting for confounding factors. Other studies have shown sex differences in the association between visceral fat and calcified atherosclerosis.^16^ Ditomasso et al suggested that visceral fat is significantly associated with arterial calcification, including the abdominal aorta and coronary arteries in men and women, and the effect of visceral fat may play a greater role in onset of atherosclerosis in women than in men.

There is one study of note that reported on the relationship between heart fat and CAC in women. El Khoudary et al^10^ assessed whether heart fat depots are significantly associated with CAC in women and concluded that greater paracardial adipose tissue volume is associated with CAC in postmenopausal women compared with premenopausal women. This previous study was, however, not based only on postmenopausal women.

The mechanisms for the association between obesity and coronary atherosclerosis remain unknown; however, estrogen deficiency might be a common mediator of both diseases among postmenopausal women. The estrogen receptor is expressed in human subcutaneous and visceral adipose tissues as well as endothelial and smooth muscle cells of blood vessels. Therefore, loss of estrogen during menopause is associated with an increase in central fat and CVD risk.^17-19^ It is, however, unknown whether menopause can affect risk factors for CVD. Our results suggest that WHR is a significant predictor of CAC. Moreover, adding WHR to the introduced clinical model improved prediction of CAC.

The present study had several limitations. First, this cross-sectional study was conducted at a single center, and the findings cannot be used to determine causal relationships. Second, our study was conducted on selective participants undergoing coronary computed tomography angiography and BIA, who visited the Health Promotion Center of our hospital. Our study was, however, conducted in healthy postmenopausal women without CVD. Third, the questionnaire had limitations in knowing the surgical history; therefore, there is a possibility that surgical menopause was included in our study. Previous studies showed that surgical menopause increased the risk of CVD.^20,21^ A population-based cohort study, however, showed that there were no significant associations between hysterectomy and CVD in women aged 50 or above, whereas hysterectomy was associated with an increased risk of CVD in women younger than 50 years.^22^ The average age of the participants in our study was over 50 years. Therefore, surgical menopause would not have a significant impact on our results. Fourth, the method used for measurement of body composition analyses is not as reliable as dual x-ray absorptiometry. The BIA method offers several advantages including ease of use and portability. Finally, history and duration of hormone therapy (HT) were not recorded in postmenopausal women. HT is known to affect the association between fat mass and coronary atherosclerosis.^10^ In spite of these limitations, this study is meaningful in that it is the first to investigate the association between body composition and coronary atherosclerosis only in asymptomatic postmenopausal women.

## CONCLUSIONS

We found that WHR, as measured by BIA was associated with CAC relative to other body composition parameters in asymptomatic postmenopausal women. Moreover, adding WHR to a clinical model improved prediction of coronary atherosclerosis. This result suggests WHR as a better marker of early atherosclerosis compared to other parameters that assess obesity status. Further prospective large-scale studies are warranted to clarify these associations.

## References

[1] Eckel RH Krauss RM American Heart Association call to action: obesity as a major risk factor for coronary heart disease. AHA Nutrition Committee. *Circulation* 1998; 97:2099 2100. 9626167 10.1161/01.cir.97.21.2099

[2] Poirier P Giles TD Bray GA Obesity and cardiovascular disease: pathophysiology, evaluation, and effect of weight loss: an update of the 1997 American Heart Association Scientific Statement on Obesity and Heart Disease from the Obesity Committee of the Council on Nutrition, Physical Activity, and Metabolism. *Circulation* 2006; 113:898 918. 16380542 10.1161/CIRCULATIONAHA.106.171016

[3] Obesity prevalence in Korea. Available at: http://www.kosis.kr/search/search.do. Accessed January 31, 2020.

[4] Yu RH Ho SC Ho SS Woo JL Ahuja AT Association of general and abdominal obesities and metabolic syndrome with subclinical atherosclerosis in asymptomatic Chinese postmenopausal women. *Menopause* 2008; 15:185 192. 17621242 10.1097/gme.0b013e31806458c9

[5] Yu JH Yim SH Yu SH The relationship of body composition and coronary artery calcification in apparently healthy Korean adults. *Endocrinol Metab (Seoul)* 2013; 28:33 40. 24396648 10.3803/EnM.2013.28.1.33 PMC3811801

[6] Lovejoy JC Champagne CM De Jonge L Xie H Smith SR Increased visceral fat and decreased energy expenditure during the menopausal transition. *Int J Obes (Lond)* 2008; 32:949 958. 18332882 10.1038/ijo.2008.25 PMC2748330

[7] Abdulnour J Doucet E Brochu M The effect of the menopausal transition on body composition and cardiometabolic risk factors: a Montreal-Ottawa New Emerging Team group study. *Menopause* 2012; 19:760 767. 22395454 10.1097/gme.0b013e318240f6f3

[8] Guthrie JR Dennerstein L Taffe JR Lehert P Burger HG The menopausal transition: a 9-year prospective population-based study. The Melbourne Women's Midlife Health Project. *Climacteric* 2004; 7:375 389. 15799609 10.1080/13697130400012163

[9] Matthews KA Crawford SL Chae CU Are changes in cardiovascular disease risk factors in midlife women due to chronological aging or to the menopausal transition? *J Am Coll Cardiol* 2009; 54:2366 2373. 20082925 10.1016/j.jacc.2009.10.009 PMC2856606

[10] El Khoudary SR Wildman RP Matthews K Thurston RC Bromberger JT Sutton-Tyrrell K Progression rates of carotid intima-media thickness and adventitial diameter during the menopausal transition. *Menopause* 2013; 20:8 14. 22990755 10.1097/gme.0b013e3182611787 PMC3528819

[11] Simon A Levenson J May subclinical arterial disease help to better detect and treat high-risk asymptomatic individuals? *J Hypertens* 2005; 23:1939 1945. 16208130 10.1097/01.hjh.0000184407.20257.58

[12] Greenland P LaBree L Azen SP Doherty TM Detrano RC Coronary artery calcium score combined with Framingham score for risk prediction in asymptomatic individuals. *JAMA* 2004; 291:210 215. 14722147 10.1001/jama.291.2.210

[13] Budoff MJ Georgiou D Brody A Ultrafast computed tomography as a diagnostic modality in the detection of coronary artery disease: a multicenter study. *Circulation* 1996; 93:898 904. 8598080 10.1161/01.cir.93.5.898

[14] Lee SN Cho JY Eun YM Song SW Moon KW Associations between osteoporosis and coronary artery disease in postmenopausal women. *Climacteric* 2016; 19:458 462. 27397609 10.1080/13697137.2016.1200550

[15] Ko BJ Chang Y Jung HS Relationship between low relative muscle mass and coronary artery calcification in healthy adults. *Arterioscler Thromb Vasc Biol* 2016; 36:1016 1021. 27034471 10.1161/ATVBAHA.116.307156

[16] Ditomasso D Carnethon MR Wright CM Allison MA The associations between visceral fat and calcified atherosclerosis are stronger in women than men. *Atherosclerosis* 2010; 208:531 536. 19765708 10.1016/j.atherosclerosis.2009.08.015

[17] Miller WL Auchus RJ The molecular biology, biochemistry, and physiology of human steroidogenesis and its disorders. *Endocr Rev* 2011; 32:81 151. 21051590 10.1210/er.2010-0013 PMC3365799

[18] Carr MC The emergence of the metabolic syndrome with menopause. *J Clin Endocrinol Metab* 2003; 88:2404 2411. 12788835 10.1210/jc.2003-030242

[19] Poehlman ET Toth MJ Gardner AW Changes in energy balance and body composition at menopause: a controlled longitudinal study. *Ann Intern Med* 1995; 123:673 675. 7574222 10.7326/0003-4819-123-9-199511010-00005

[20] Punnonen R Ikalainen M Seppala E Premenopausal hysterectomy and risk of cardiovascular disease. *Lancet* 1987; 1:1139. 10.1016/s0140-6736(87)91689-8 2883456

[21] Rosenberg L Hennekens CH Rosner B Belanger C Rothman KJ Speizer FE Early menopause and the risk of myocardial infarction. *Am J Obstet Gynecol* 1981; 139:47 51. 7457520 10.1016/0002-9378(81)90410-5

[22] Ingelsson E Lundholm C Johansson AL Altman D Hysterectomy and risk of cardiovascular disease: a population-based cohort study. *Eur Heart J* 2011; 32:745 750. 21186237 10.1093/eurheartj/ehq477

